# A review on delayed toxic effects of sulfur mustard in Iranian veterans

**DOI:** 10.1186/2008-2231-20-51

**Published:** 2012-10-09

**Authors:** Seyed mansour Razavi, Payman Salamati, Masoud Saghafinia, Mohammad Abdollahi

**Affiliations:** 1 Department of Community Medicine, Tehran University of Medical Sciences, Tehran, Iran; 2 Sina Trauma and Surgery Research Center, Tehran University of Medical sciences, Tehran, Iran; 3 Trauma Research Center, Baqiyatallah University of Medical Sciences, Tehran, Iran; 4 Department of Toxicology and Pharmacology, Faculty of Pharmacy and Pharmaceutical Sciences Research Center, Tehran University of Medical Sciences, Tehran, 1417614411, Iran; 5 Sina Trauma and Surgery Research Center, Sina Hospital, Hassan Abad Square, Imam Khomeini Avenue, Tehran, Iran

**Keywords:** Chemical injuries, Chemical victim, Chemical warfare agents (CWA), Sulfur mustard, Mustard gas, Toxic effects of sulfur mustard

## Abstract

Iranian soldiers were attacked with chemical bombs, rockets and artillery shells 387 times during the 8-years war by Iraq (1980–1988). More than 1,000 tons of sulfur mustard gas was used in the battlefields by the Iraqis against Iranian people. A high rate of morbidities occurred as the result of these attacks. This study aimed to evaluate the delayed toxic effects of sulfur mustard gas on Iranian victims. During a systematic search, a total of 193 (109 more relevant to the main aim) articles on sulfur mustard gas were reviewed using known international and national databases. No special evaluation was conducted on the quality of the articles and their publication in accredited journals was considered sufficient. High rate of morbidities as the result of chemical attacks by sulfur mustard among Iranian people occurred. Iranian researchers found a numerous late complications among the victims which we be listed as wide range of respiratory, ocular, dermatological, psychological, hematological, immunological, gastrointestinal and endocrine complications, all influenced the quality of life of exposed victims. The mortality rate due to this agent was 3%. Although, mortality rate induced by sulfur mustard among Iranian people was low, variety and chronicity of toxic effects and complications of this chemical agent were dramatic.

## Background and history

Mustard gas or sulfur mustard is a chemical warfare agent (King of the battle gases) [1] with cytotoxic, vesicant and blistering effects on exposed skin [2]. This agent can enter the body through various routes including the skin, the respiratory system, conjunctiva and the gastrointestinal system by contaminated foods [3]. It can cause both acute and delayed manifestations and late complications even 40 years after the exposure as reported for victims of the first worldwar [4]. People can be exposed to small or large amounts of mustard gas through terroristic actions, wars, leakage from the factories, and even activities like fishing [5].

Sulfur mustard can be easily produced. It has a delayed, extensive absorption, multi-organ effects and quick penetration. It is stable in the environment, has a low production cost, is easy to use and has the ability to debilitate soldiers [6,7]. After dissemination in an area, mustard gas remains in that area for a long time and makes that area non-habitable. Mustard gas victims temporarily lose their vision a few hours after exposure which is a critical strategy in warfare [7], and so far, it has been used in >10 military conflicts [8].

Human losses related to destructive effects from this agent in the World War l are as follow: the soldiers were only equipped with protective breathing masks and lack of skin protection resulted in the death of more than 90,000 soldiers and about 1.3 million injured people [8] and out of which, 400,000 people required long term medical care [9]. According to the other reports mustard gas caused 14,000 injuries in the first 3 months of use in World War I and a total of 120,000 injuries [10]. In the US army, out of the total of 36,965 chemical warfare victims, 27,711 (75%) were due to sulfur mustard gas, and according to a report from the contamination control unit of the British army, of a total of 160,970 chemically injured soldiers, 124,752 (77.5%) had been injured by sulfur mustard gas. The greatest damage by chemical warfare agents in World War I was done to the Russians resulting in 50,000 casualties and 500,000 injuries. Years after the application of mustard gas in World War I, its delayed progressive and destructive effects were recognized [7].

In the beginning of Iraq-Iran war in 1980, use of chemical warfare agents was limited but in March 1985, despite international conventions on prohibition of using chemical warfare agents, Iraqis extensively used these agents against Iran. They used nerve agents (Sarin & Tabun) and mustard gas against Iranian soldiers [11,12].

According to data reported by Veterans and Martyrs Affair Foundation (VMAF) which is responsible for taking care of the war victims in Iran, the Iranian people were 387 times attacked with chemical bombs, rockets and artillery shells during the 8-years war by Iraq (1980–1988) [13]. More than 1,000 tons of sulfur mustard was used in the battlefields [2] and about 100,000 of people were injured due to this agent [8], as at the present, after more than two decades, still about 30,000 of them are under treatment [14].

Sardasht is a city in north-west of Iran. Iraqis released four 250 kg bombs containing mustard gas on this city at 4 pm, July 27, 1987 injuring 4,500 civilians [15]. Use of mustard gas against Iranian soldiers by the Iraqis was reported to the international commissions by Iran in 1984 and in 1986 application of this agent by Iraqis was documented by the United Nations observatory team led by M. Dominguez [16]. They evaluated the battlefields, clinically examined the victims and performed some para-clinical tests and approved that Iraqis had used chemical bombs containing mustard gas and to a lesser extent organophosphorous nerve agents especially Tabun [17].

Mustard gas was used by the Iraqis in south and west of Iran many times and in 1988 the last chemical attack was performed on Oshnaviyyeh city injuring 2,680 civilians [13-18].

### Definition and etiology

When mustard plants with the Latin name, Synapsis is affected by the tyrosine glycoside Synacrine enzyme, a substance is produced that naturally does not exist in the seeds of this plant. This compound is isothiosyanate which it is a vesicant agent [19]. Mustard compounds are classified among the alkylating agents that substitute hydrogen with an alkyl cation. A large group of these compounds such as nitrogen mustards (chlorambucil) is mainly used in medicine for treatment of cancers. The other group namely sulfur mustard is considered a chemical warfare agent used in many battlefields in the 20^th^ century [3]. This agent is recognized with various abbreviations H, HD, HT, LOST, SM (sulfur mustard), MG (mustard Gas) and Y perit “H” and “HD” come from the words “Hun Stuffe” and “Distilled”, HT is a combination of 6% HD and 40% of a substance called T and the LOST come from the name of the scientists Lommel and Stein kopf, who developed a method for the large-scale production of the agent for the German army. French say to this agent, Y perit because it was used in the Y pres at the first time [20].

Tables 1 and 2 indicate the history and properties of sulfur mustard based on literature [1,3,6-10,18,21-26].

**Table 1:** Historical evolution

**Year**	**Event**	**Reference no.**
1822	A type of Mustard gas was developed by César-Mansuete Despretz, the Belgian scientist.	[8]
1860	Frederick Guthrie noted its blistering properties	[1,8]
1886	Victor Meyer produced pure sulfur mustard.	[1]
1917-1918	First use in World War I by the German army - along 10 days about 1 million sulfur mustard shells were poured on Belgian soldiers. 1.3-2.5 million people in Belgium and France and thousands of British soldiers were injured.	[1,8,10]
1919	Production of mustard gas in American factories reached 19 tons a day.	[7]
1925	Use of these agents was banned in Geneva Gas Protocol.	[8]
1935- 1936	Italy breached the Geneva Protocol treaty and used sulfur mustard gas against Ethiopia when conquering the Ethiopian plane.	[8]
1943	A cargo ship carrying a large amount of sulfur mustard exploded in the harbor of Bari, Italy. The gas was disseminated in the area injuring more than 600 people.	[1]
1937-1945	Japan used sulfur mustard gas against China.	[8,10]
1945-1948	A large amount of sulfur mustard was poured in the Baltic Sea. Exposure of Scandinavian fishermen to this agent resulted in development of skin blisters	[1]
1963-1967	Egypt used sulfur mustard bombs against Yemeni pro-monarchy supporters.	[1,8]
1983-1988	Iraq extensively used SM and nerve agents against Iran and injured more than 50,000 victims.	[8,9]
1988	Iraqi army killed 5,000 Iraqi civilians in Halabcheh using SM. In this attack, nerve agents like sarin were also used.	[1,8]

**Table 2:** Physical properties of sulfur mustard

**Properties**	**Description**	**References**
**Formulation**	C4H8Cl2S	[1,18]
	2 side chains of dichloroethyl/sulfide or bis (2-chloro-ethyl) sulfide	
**Color**	Colorless, lucent, or pale yellow (pure) to yellow, brown, dark brown or black color (impure).	[8,18]
**Odor**	Sulfur mustard has a slight garlic, horseradish, addled egg or fried vegetables or mustard type odor.	[8,14,18]
**Form**	Oily substance, liquid (in room temperature), solid, powder (decontamination is much more difficult), gas or vapor. It transforms into aerosols in 105°C.	[3,8,18,21]
**Chemical reaction**	Neutral	[8]
**Solubility**	Lipophilic substance and highly fat soluble (it can easily disseminate into skin, mucosa, brain, kidneys, muscles and liver), negligible solubility in water, may be hydrolyzed in water and soluble in acetone. Water solubility 0.092 g/100 g at 22°C.	[3,8,22]
**Stability**	In low temperatures, sulfur mustard remains stable in clothing and soil for months. It remains in the battlefields (for example beside the moats in World War I) and can be found in the amount of 1–25 mg/m3 in 6–12 inch depth of the soil. In moderate temperatures with mild winds sulfur mustard can remain stable for more than a week . Different forms of SM can be stored in the soil for up to 10 years.	[8,23]
**Boiling point**	215 - 227°C	[1,8]
**Melting point**	13-14°C	[1]
**Freezing point**	14°C (its freezing point is decreased by chlorobenzene)	[8]
**Volatility**	610 mg/m3 in 20°C	[8]
**Specific Gravity**	1.27(in liquid form than water and in gas form is heavier than air)	[21,24]
**Vap. Pres.**	0.072 mmHg at 20°C	[8]
**Molecular weight**	159.08	[1]
**Evaporation degree**	SM evaporates at 15°C, in warm temperatures becomes less stable and its vapor form increases, and at night it sediments because of decreased temperature.	[7]
**Density**	SM is heavier than water when in the form of liquid and heavier than air when in the form of vapor or gas. Liquid density (1.274g/ml, Vapor Density (5.4), Solid Density (Crystal) 1.37 g/ml at 20°C	[1,8]
**Half life**	5 min in 37°C	[3]
**Permeability**	SM penetrates the porous clothing and food and plants, easily penetrates into the cell membrane of most tissues, wood, leather (it an permeate leather and regular clothing in a few minutes and reach body tissues), rubber, plastics (can easily pass through regular or plastic breathing masks and rubber or plastic clothing can protect the body for a few hours) and remains active for a long time in cold or moderate temperatures.	[7,23,25]
**Absorption in the body**	Eighty percent of the SM gas is evaporated and the remaining 20% penetrates the body. Of the 20%, 12% remains on the skin and 8% is absorbed systemically. Absorption is done through moist tissues like respiratory system, axillary area, genital area/groins and eyes. Tissues with higher metabolism are more sensitive to this toxic gas.	[1,3,8,26]
**Metabolites**	The main metabolite of SM in the urine is thiodiglycol which can be detected by chromatography with1ng /ml sensitivity.	[3]
**Excretion**	Fifty percent of the absorbed SM in the body is conjugated with aminoacid lecithin producing di-cystylethyl sulfone which is excreted through the kidneys.	[22]
**Anti-toxin**	Unfortunately there is no specific antitoxin for SM gas	[6]
**Applications**	Except for chemical warfare, a nitrogen analogue of SM is now being used in chemotherapy for treatment of leukemia. This therapeutic agent is called Mustargen	[1]

### Pathology and mechanisms of actions

In summary, this alkylating substance leads to DNA damage, cell membrane damage, decrease in glutathione, activation of nuclear factor kappa-light-chain-enhancer of activated B cells (NFkB), and caspase activation. DNA damage lead to Poly (ADP-ribose) polymerase (PARP) activation and nicotinic adenine nucleotide (NAD) depletion and this process in turn will lead to necrosis. Glutathione decreasing produce reactive oxygen and these two phenomena may lead to necrosis and cell death [27]. In addition to cell death, SM has many other adverse effects on cells such as alkylation effects, mitosis inhibition (effects on hematologic system, immunologic system, epithelial and germinal tissues), mutagenesis, carcinogenesis, and colinomimethic effects [28].

On the basis of Kehe et al. study, in summary, mustard gas in the molecular level induces the releasing of cytokines, prostaglandines, matrix metalloproteinases (MMPs) and serine proteases, and increases DNA damages, oxidative stress, and impaired energy metabolism. Following to these molecular changes, cellular infiltration, apoptosis and necrosis occur that is continued by erythema and pain and formation of vesicles, blisters, ulcer and impaired wound healing [27].

Regarding above mechanisms, some authors suggeste use of antioxidants, antiinflammatoriy, PARP inhibators and N-acethyl-cystein (NAC) in treatment of SM toxicity.

## Methods

During a systematic search, a total of 193 medical articles related to SM were reviewed using known international medical databases such as Scopus, Medline, ISI, and Iranian medical databases such as Iranmedex, SID, and Irandoc. One hundred and nine articles were more relevant to the main aim. Eight articles were on general aspects of SM effects, 36 articles were related to respiratory effects, 16 articles were on dermatologic effects, 15 articles were on ophthalmologic effects, 11 articles on psychological effects, 10 articles on endocrinology & reproductive health effects, 4 articles related to quality of life and 9 articles were related to other items such as: neurologic, oncologic, hematologic, cardiologic, laboratory. No special evaluation was conducted on the quality of the reviewed manuscripts and the credit of journal was considered sufficient. Our study had been handled in accordance with the rules of the ethical review board of Tehran University of medical sciences.

## Results

### Initial description of victims

Some Iranian soldiers described their first encounter with SM as follows: immediately after exposure to the SM bomb, we smelled a garlic odor and had a bitter taste in our mouth. A few hours later, we became dizzy, with a headache and we could not breathe. A short time after the explosion, we developed small bleeding areas and inflamed small lesions (macules and papules) on our skin. Then we felt hoarseness in our voice and this condition has been going on and off so far [28,29]. Also, some other victims described the events at the exposure time as follows:

We were exposed to SM gas delivered through bomb explosion from 5 to 30 meters distance. We smelled a strong smell of garlic, addled egg or fried vegetables. A bluish-grayish cloud and a white dust appeared in the sky. We had no protection. In the first hours following the attacks, we washed our faces and hands with waters which we did not know the water may be contaminated with SM [14]. Gradually, ocular, dermatologic and respiratory symptoms developed. We are still suffering from the related respiratory symptoms.

Mustard effects on body organs are divided into acute and chronic phases. Most of the reviewed articles were performed in chronic phase of exposure which they are summarized in Table 3.

**Table 3:** Distribution of pulmonary, ophthalmic and dermatologic complications in several studies in Iran

**Reference**	**First author**	**After exposure time (years)**	**Agent**	**Under studied population**	**Number**	**Ocular complications (%)**	**Pulmonary complications (%)**	**Dermatologic complications (%)**
[30]	**Khateri**	13 - 20	Mustard	Veterans	34000	93.3	42.5	24.5
[30]	**Khateri**	14	Mustard	Children	50	86	100	98
[31]	**Ghassemi Broumand**	19	Mustard	Civilian population	600	37.7	45.8	31.5
[32]	**Balali Mood**	3 - 9	Mustard + Nerve agents	Veterans	1428	88	90	78
[10]	**Ghassemi Broumand**	17 - 22	Various chemical agents	Militaries + civilian	479	26	32.1	23.3
[33]	**Etezad Razavi**	16 - 20	Mustard	Veterans	40	65	95	90
[15]	**Ghanei**	15	Mustard	Civilian population	108	*	100	*
[34]	**Emadi**	14 - 20	Mustard + Nerve agents	Veterans	800	*	*	100

Percentage of organ involvement has been reported differently in various Iranian studies. For example, Balali and colleagues (1992) in their study evaluated the delayed toxic effects of mustard gas on 1,428 chemical victims 3–9 years after exposure and reported the most prevalent complications to be respiratory complications (90%), dermatologic complications (88%), ocular complications (78%), neural complications (71%), GI complications (55%), genital complications (52%) and hematopoietic system complications (38%) [32].

In a study conducted on 100 Iranian chemical warfare victims, 94% had dermatologic, 94% had ophthalmic, 75% had pulmonary, 5% had GI and 10% had hematologic complications as the result of chemical exposure [16].

In another study, delayed toxic effects of mustard gas were evaluated in 236 Iranian chemical victims 2 to 28 months after exposure and complications were as follows: respiratory complications in 78%, CNS in 45% and dermatologic complications in 24.5% of cases [35].

Table 3 shows distribution of pulmonary, ophthalmic and dermatologic complications in several studies in Iran [10,15,30-34].

According to report of Khateri and colleagues, the pulmonary, ophthalmic and dermatologic complications were the most common in 34,000 victims observed [30].

### Complications

Mustard gas complications in Iranian victims were as follows:

### Respiratory system complications among Iranian Veterans include

Obstruction of upper airways, chronic bronchitis, bronchiolitis, bronchiectasis [10,36], asthma [2,10], COPD [37,38], emphysema, stenosis of large airways, pulmonary fibrosis [10,39], thickening of the bronchial walls, air trapping, bronchiolitis obliterans organizing pneumonia (BOOP) (a type of chronic pneumonia) [40], chronic laryngitis and hoarseness of voice (acid like burning sensation) [29], rhinopharyngitis, paranasal sinuses involvement, tracheobronchitis [39], laryngeal carcinoma [10], lung cancer in continuous exposure [18], tracheobronchomalacia [41], airway collapse [18] and recurrent respiratory infections and acute respiratory failure [1,2]. The main cause of death in the soldiers who died immediately on the battlefield, was probably pulmonary edema [29].

### Ocular complications among Iranian victims were as follows

Eyelid edema, blepharospasm, chemosis, conjunctivitis, limbal ischemia, limbal pigment loss, limbal loss, retina ulcer, blurred vision, visual impairment, scarring and neovascularization of the anterior chamber, uveitis, corneal opacity, keratitis, corneal melting, calcium deposition, conjunctivalization, perforation, and rarely blindness [4,7,10].

### Dermatologic complications among Iranian injured people were

Chronic dryness and pruritus, burning sensation, increased sweating, hair loss, observation of erythema, macular and popular rashes, vesicles, pustules, blisters, ulcers, plaques, urticaria and angioedema, mustard scars, cloid, hyper pigmentary, hypo and depigmentary lesions, skin atrophy, lickenification, excoriation, scaling, neurosis, acne form lesions, melanocytic nevi, telangiectasia, cherry angiomas, seborrhea dermatitis, tinea versiculer, keratosis pillars, vitelligo, alopecia areata, liken planus, actinic keratosis, and multiple basal cell carcinomas [10,21,42-48].

### Psychological complications among the Iranian victims

According to one study based on general health questionnaire, which were conducted on 206 victims from Sardasht city (a city which were on direct attacks), 95.1% of them, did not have a healthy psychological status [10]. Other information about the psychological disorders were: anxiety 15%, depression 46%, personality disorders 31%, psychosis 3% [32], post-traumatic stress disorder (PTSD) [10], “disorders of consciousness (2%), attention (54%), emotion (98%), behavior (80%), thought process (14%), and memory (80%) were reported, 3–5 years after exposure, by Tabatabai and colleagues” [1] and decrease of self-steam [49]. Madarshahian has reported some changes in current life among exposed patients as follows: irregular sleep (55%), nightmare (15%), using tobacco (60%), depression (40%), bad mood and anger (40%), lack of interest in social activities (44%), loss of confidence (55%), paranoia (45%), nervousness (46%) [50].

### Genital complications include

Sexual dysfunction, erectile dysfunction, impotence, decreased libido, infertility, spermatological disorders, miscarriage in victims wives, premature ejaculation, congenital disorders in the newborns, late ejaculation or inability to ejaculate [3,51].

### Hematologic complications include

Leukopenia in the first days following exposure, decreased T cells, anemia, thrombocytopenia [7], leukemia [21] and hormonal changes [52].

### Neurotoxicity include

Sever exposure to SM affects the CNS leading to convulsion in animal [53] as neurotoxicity (delayed neuropathy syndrome) or chronic neuropathic pains has been reported [16-30,32,35]. SM can cause [30]. Balali and colleagues performed electromyography (EMG) and nerve conduction velocity (NCV) on 40 Iranian soldiers and witnessed delayed peripheral neuropathy in 77.5% of cases [1]. In a study on 100 chemical victims, those who were severely disabled had 4.09 times greater risk of developing neuropathy compared to those with moderate or mild disability. NCV and EMG tests revealed that 5% of all under studied victims had axonal neuropathy [16].

### Other complications include

Decreased quality of life and sleep disorders as the result of several physical (ophthalmologic injuries, pruritus, COPD and …) and mental complications [9,54], contamination of the wives of exposed soldiers [3,55]. Mustard gas also can cause liver and kidney failure, skin cancer, and bone marrow depression [56]. McNamara and colleagues in their study on rats found that using 0.5 to 2 mg/kg mustard gas through the stomach tube in days 6 and 15 of pregnancy caused no evidence of teratogenicity [15,57].

The most prevalent GI complications in Iranian victims were nausea, vomiting, loss of appetite, stomach pain and diarrhea. Mustard gas can cause stomach cancer, basal cell carcinoma, Bowen’s carcinoma and spinocellular carcinoma [1]. Mustard gas can eventually cause death. However, the mortality rate is low (2 – 3%) [8,58].

## Discussion

According to initial description of the victims, there is a latent phase between the exposure and appearance of symptoms. This time interval increases the morbidity of exposed victims. Victims at first are not aware of their exposure/contamination and as the result they do not attempt to clean themselves which consequently results in greater absorption of the toxic agent into the tissues. This phase usually takes between 30 minutes to 8 hours [8].

To deal with this phase soldiers should train on how to deal with chemical terrorism, especially with mustard gas. For example, they should avoid washing their hand, face and body with water in the area that was attacked because it may be contaminated with the poison.

Longitudinal studies showed that those exposed to mustard gas suffer from long term complications causing significant morbidity [8]. There are 2 types of complications: early and late. Early complications appear in the first week following exposure but late complications may manifest in the next 50 years following exposure [10]. In chronic phase, patients may be suffering from various diseases.

During World War I, scientists found that mustard gas can cause bone marrow aplasia, dissolution of lymphoid tissue and GI ulcers. Later many more complications were reported due to mustard gas including respiratory, dermatologic, ocular, GI, hematopoietic, endocrine, neural, psychological, ear-nose-throat (ENT), genital, reproductive and immune system [21] involvement which can manifest as early or delayed complications.

In Iranian studies, all the above mentioned effects have been reported. Moreover, there are some studies about sleep disorders [59], immunologic disorders [1,60], oncologic diseases [61], cardiologic diseases [62], exposed military and civilian rights [63], abnormal lab examination results [64] and death among Iranian victims [56].

Mustard gas is a chemical warfare agent that is detrimental for the earth and has a significant toxic effect on microorganisms in the soil. It also inhibits the enzymatic activity of the soil [65]. On the other hand, SM can remain in the battlefields (for example beside the moats in World War I) and can be found in the amount of 1–25 mg/m3 in 6–12 inch depth of the soil. In moderate temperatures with mild winds SM can remain stable for more than a week [8]. Different forms of SM can be stored in the soil for up to 10 years [10]. Considering all the above facts, since the war zones in our country now are major tourist areas, and every year a large number of people travel to these areas, healthy soil-building, should strictly be placed on the agenda of the popular visits.

There are different reports about the prevalence of death and its causes in Iranian veterans. Ghanei et al., in their study have stated that, the rate of death among 1,005 people who died during their study period, were 72% by diseases, 16.1% due to unintentional accidents, 2.3% due to suicide, and 0.8% caused by intentional accidents. The causes of deaths in 2.8% were uncertain and 12.1% of deaths were related to chemical agents effects [66].

Tavallai et al., in their study conducted on 1239 Iranian veterans also reported that 65.4% of deaths were due to diseases, and 24.7% were due to accidents [67].

In summary, reported fatality rates in exposed soldiers in World War 1 were less than 2% and among Iranian victims were 3-4% and disabling potential of SM is much greater than lethality of this agent [68]. According to Tavallai report, the most common causes, had led to death were cardiac diseases and this according to an other report, in Iranian victims 75% of deaths were due to respiratory complications [18], and many of Iranian soldiers lost their lives in a chemical battle scene and probably the cause of death was pulmonary edema induced by chemical agents (29).

## Conclusion

Although, mortality rate induced by sulfur mustard among Iranian people was low, the variety and chronicity of adverse effects and complications of this chemical agent were dramatic.

## Competing interests

The authors declare that they have no competing interests.

## Authors contributions

SMR theorized, designed and interpreted the data and drafted the manuscript. PS abstracted, designed, acquainted the data and revised the manuscript. MS theorized the data, drafted and revised the manuscript. MA revised data and revised the manuscript. All authors read and approved the final manuscript.
